# Influence of Fucoidan Extracts from Different Fucus Species on Adult Stem Cells and Molecular Mediators in In Vitro Models for Bone Formation and Vascularization

**DOI:** 10.3390/md19040194

**Published:** 2021-03-29

**Authors:** Fanlu Wang, Yuejun Xiao, Sandesh Neupane, Signe Helle Ptak, Ramona Römer, Junyu Xiong, Julia Ohmes, Andreas Seekamp, Xavier Fretté, Susanne Alban, Sabine Fuchs

**Affiliations:** 1 Experimental Trauma Surgery, Department of Orthopedics and Trauma Surgery, University Medical Center Schleswig-Holstein, 24105 Kiel, Germany; fanluwang@gmail.com (F.W.); xiao_yuejun@163.com (Y.X.); ramona.roemer@googlemail.com (R.R.); serene.xj@gmail.com (J.X.); Julia.Ohmes@uksh.de (J.O.); Andreas.Seekamp@uksh.de (A.S.); 2 Department of Pharmaceutical Biology, Pharmaceutical Institute, Kiel University, 24148 Kiel, Germany; sneupane@pharmazie.uni-kiel.de (S.N.); salban@pharmazie.uni-kiel.de (S.A.); 3 SDU Chemical Engineering, University of Southern Denmark, 5230 Odense, Denmark; sihp@igt.sdu.dk (S.H.P.); xavier.frette@gmail.com (X.F.)

**Keywords:** fucoidan, mesenchymal stem cells (MSC), outgrowth endothelial cells (OEC), angiogenesis, bone vascularization

## Abstract

Fucoidans, sulfated polysaccharides extracted from brown algae, are marine products with the potential to modulate bone formation and vascularization processes. The bioactivity and safety of fucoidans are highly associated with their chemical structure, which may vary with algae species and extraction method. Thus, in depth evaluation of fucoidan extracts in terms of endotoxin content, cytotoxicity, and their detailed molecular biological impact on the individual cell types in bone is needed. In this study, we characterized fucoidan extracts from three different *Fucus* species including *Fucus vesiculosus* (Fv), *Fucus serratus* (Fs), and *Fucus distichus* subsp. *evanescens* (Fe) for their chemical features, endotoxin content, cytotoxicity, and bioactive effects on human outgrowth endothelial cells (OEC) and human mesenchymal stem cells (MSC) as in vitro models for bone function and vascularization. Extracts contained mainly high molecular weight (HMW) fucoidans and were free of endotoxins that may cause inflammation or influence vascularization. OEC tolerated fucoidan concentrations up to 200 µg/mL, and no indication of cytotoxicity was observed. The inflammatory response, however, investigated by real-time PCR (RT-PCR) and enzyme-linked immunosorbent assay (ELISA) and endothelial barrier assessed by impedance measurement differed for the individual extracts. MSC in comparison with endothelial cells were more sensitive to fucoidans and showed partly reduced metabolic activity and proliferation at higher doses of fucoidans. Further results for MSC indicated impaired osteogenic functions in alkaline phosphatase and calcification assays. All tested extracts consistently lowered important molecular mediators involved in angiogenesis, such a VEGF (vascular endothelial growth factor), ANG-1 (angiopoietin 1), and ANG-2 (angiopoietin 2), as indicated by RT-PCR and ELISA. This was associated with antiangiogenic effects at the functional level using selected extracts in co-culture models to mimic bone vascularization processes during bone regeneration or osteosarcoma.

## 1. Introduction

Fucoidans, sulfated polysaccharides derived from brown algae, have been reported to influence a series of physiological key processes such as inflammation [1,2,3], vascularization [4,5], as well as the recruitment of stem cells [6]. All these mentioned biological processes play a fundamental role for a variety of different tissues or diseases and are of particular interest for applications in bone health, ranging from the maintenance of functional bone tissue and enhancement of bone regeneration and repair, up to the treatment of bone tumors, such as osteosarcoma [7,8,9].

In most cases, extracts comprise a heterogeneous composition with large variations in monosaccharide composition, molecular weight, sulfation degree, etc. [10,11]. Accordingly, the structure–function relationship for fucoidans often remains unclear, thus limiting their suitability as a biomedical compound. One key prerequisite for using fucoidans in biomedical applications is to develop purified and standardized extracts with well-defined chemical characteristics [12]. In addition to the production of extracts with distinct physical or chemical properties, the evaluation of the tissue- or cell type-specific effects is an important strategy to ensure biological safety by selecting non-toxic but bio-functional doses [13,14]. Further, tests of fucoidans in selected cell models providing controlled physiological conditions can contribute to a better understanding of how fucoidans modulate processes, such as bone formation or vascularization. In addition, such tests enable identification of the associated molecular mediators influenced by fucoidan extracts. In previous reports, we have demonstrated biological effects in in vitro models for bone and associated vascularization processes using a crude fucoidan extract from *Fucus vesiculosus* [4] or fractionated extracts from *Fucus distichus* subsp. *evanescence* [15]. Before biomedical use of fucoidans, complex testing procedures are necessary. This is a challenging task, especially when considering the large number of extracts that can be gained from the different species of brown algae, as well as from different extraction methods or processing technologies, such as fractionation [10,16,17]. In this context, a detailed characterization of the molecular biological effects of fucoidans in the target cells will support the selection of extracts with the best biomedical effect for the envisioned therapeutic aim, contribute to their biological safety, and enable definition of structural and functional relationships at cellular and molecular levels.

In this study, using a variety of characterization methods in in vitro models, we prepared and tested fucoidan extracts from different *Fucus* species and compared them in terms of their chemical characteristics, endotoxin content, and their biological impact on endothelial cells and bone-forming mesenchymal stem cells.

## 2. Results

### 2.1. Chemical Characterization of Fucoidan Extracts

Analysis of the monosaccharide composition by acetylation analysis of the six fucoidans from *F. vesiculosus* (Fv), *F. distichus* subsp. *Evanescens* (Fe), and *Fucus serratus* (Fs) revealed that Fv3, Fe, and Fs1 had the highest fucose content (above 75%) along with the highest degrees of sulfation (DS), whereas the other three extracts had a fucose content below 60%. In addition, Fv1 and Fs2 contained a considerable amount of glucose (Table 1), which probably represents co-extracted ß-1,3-glucans of brown algae.

The molecular mass (MW) of the fucoidans largely differed from 84 kDa (Fe) to 730 kDa (Fv2). The MW and size parameter of the samples are summarized in Table 1. Further, the MW distribution plots of the fucoidans and MW ranges of three weight fractions (≤20%, 20–80%, ≥80% (*m/m*)) of different fucoidans are included as Supplementary Materials (Table S1, Figures S1 and S2). These data illustrate that all the fucoidans extracts represent highly polydisperse mixtures; even the MW of Fe with the lowest polydispersity ranged from 22 kDa to 403 kDa. 

### 2.2. Endotoxicity 

Before characterizing the cytotoxicity and biological activity, the endotoxin content in the extracts was determined by EndoLISA to exclude influences by endotoxins that might further cause endothelial cell activation and severe inflammatory responses. As depicted in Table 2, endotoxin levels were below critical values for all tested extracts in this study. 

### 2.3. Effect of Fucus Extracts on Metabolic Activity and Cytotoxicity in Endothelial Cells 

**Figure 1:**
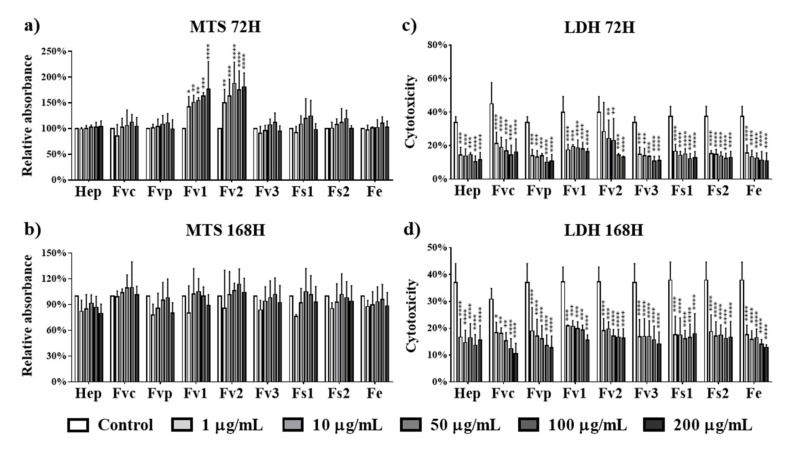
Cell metabolic activity of outgrowth endothelial cells (OECs) determined by MTS assay at (**a**) 72 h and (**b**) 168 h and cytotoxicity for OECs determined by lactate dehydrogenase (LDH) at (**c**) 72 h and (**d**) 168 h after treated with fucoidan extracts and heparin at different concentrations of 1, 10, 50, 100, and 200 µg/mL. Two-way ANOVA, * *p* < 0.05, ** *p* < 0.01, *** *p* < 0.001, **** *p* < 0.0001, *n* = 3.

Independent from *Fucus* species and the tested concentration (1 to 200 µg/mL) (Figure 1), no cytotoxic effects were observed for tested extracts in human microvascular endothelial cells from the peripheral blood (OEC). Using lactate dehydrogenase (LDH) assessment as an indicator for cell membrane damage, all the tested extracts resulted in reduced LDH levels observed at both investigated time points (72 h, 168 h). Remarkably, in contrast to the other test compounds and to the results on day 7, Fv1 and Fv2, the two fucoidans with the highest MW but lowest DS, significantly improved the metabolic activity on day 3. In agreement with the test principles of MTS and LDH, assay data resulted in complementary results for metabolic activity and cytotoxicity.

### 2.4. Impact of Fucus Extracts on the Endothelial Barrier

**Figure 2:**
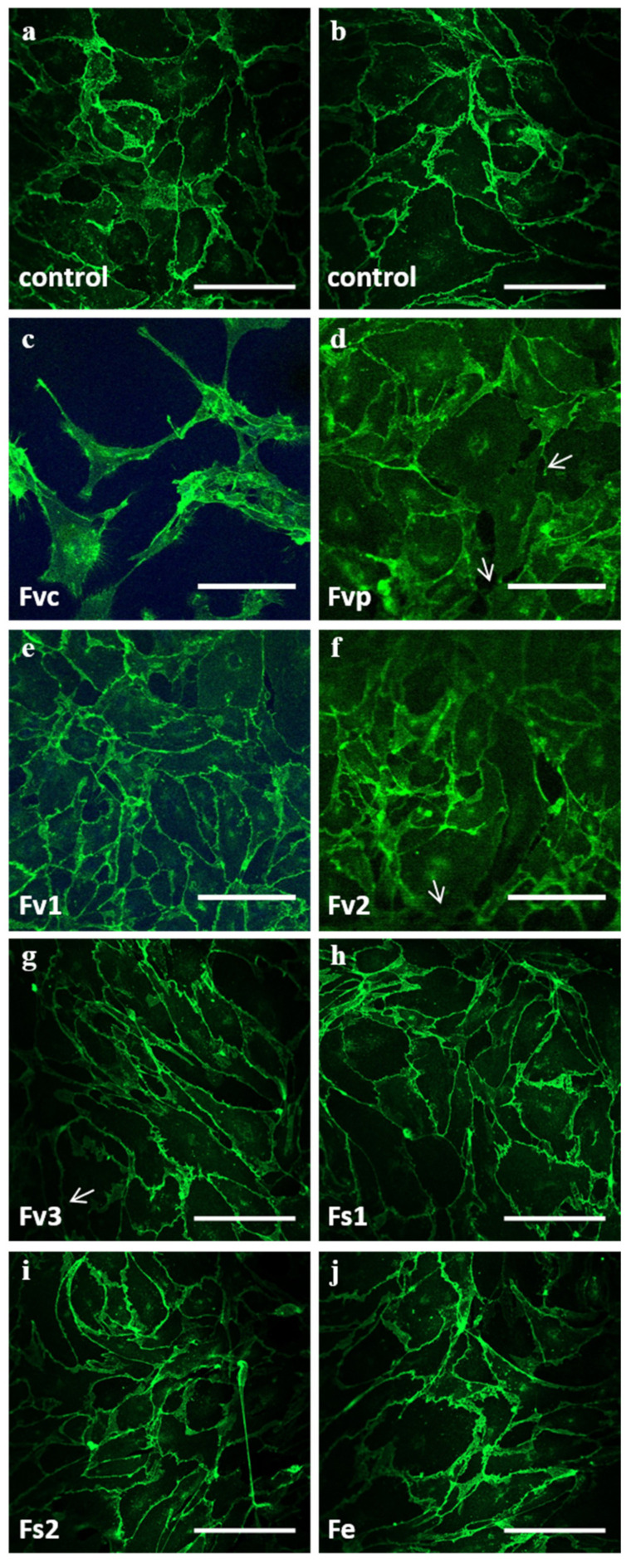
Confocal laser scanning microscopy of OEC mono-cultures on day 7 for (**a**,**b**) controls and treatment of 100 μg/mL fucoidan extracts: (**c**) Fvc, (**d**) Fvp, (**e**) Fv1, (**f**) Fv2, (**g**) Fv3, (**h**) Fs1, (**i**) Fs2 and (**j**) Fe. VE-cadherin is depicted in green. The scale bar represents 100 μm.

Morphological assessment of OEC by immunofluorescence for VE-cadherin after seven days of treatment (100 µg/mL) indicated partly a rearrangement of the cell layer and the endothelial cell–cell contact mediated barrier. This effect was most prominent for the commercially available crude fucoidan from *Fucus vesiculosus* (Figure 2). Morphological assessment further indicated to some extent effects on the cell–cell contacts for other *Fucus vesiculosus* extracts (Figure 2, arrow); these, however, were much less evident.

**Figure 3:**
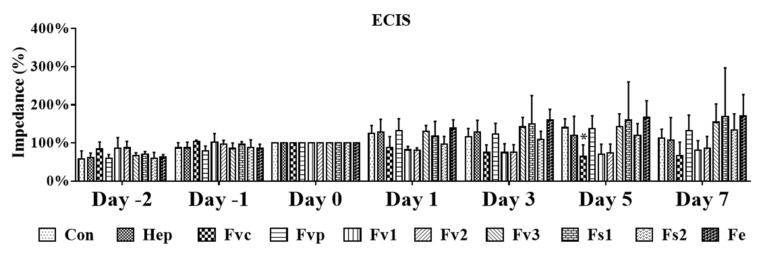
Cell barrier characterized by electrical cell-substrate impedance sensing (ECIS) for OEC mono-culture in response to fucoidan and heparin at 100 µg/mL treatment. Two-way ANOVA, * *p* < 0.05, *n* = 3.

To quantify potential effects of the tested fucoidans on the endothelial barrier, we performed electric impedance measurements using an electrical cell-substrate impedance sensing (ECIS) system (Figure 3). For this, OEC were grown until the impedance reached a stable level as an indicator of a stable and tight endothelial cell layer. Then, OEC were treated with the fucoidan extracts for a further seven days. Depicted impedance values were normalized to measured values on day 0 (100%). In accordance with the morphological observation in Figure 2, treatment with the crude fucoidan from *Fucus vesiculosus* resulted in a significant reduction of the impedance on day 5. A similar effect was observed for Fv1 and Fv2, although here the reduction of the impedance showed only a tentative trend. In contrast, the barrier of OEC after treatment with Fs1, Fs2, and Fe remained stable, and no impedance reduction was observed.

### 2.5. Effects of Fucus Extracts on Endothelial Activation and Inflammation 

**Figure 4:**
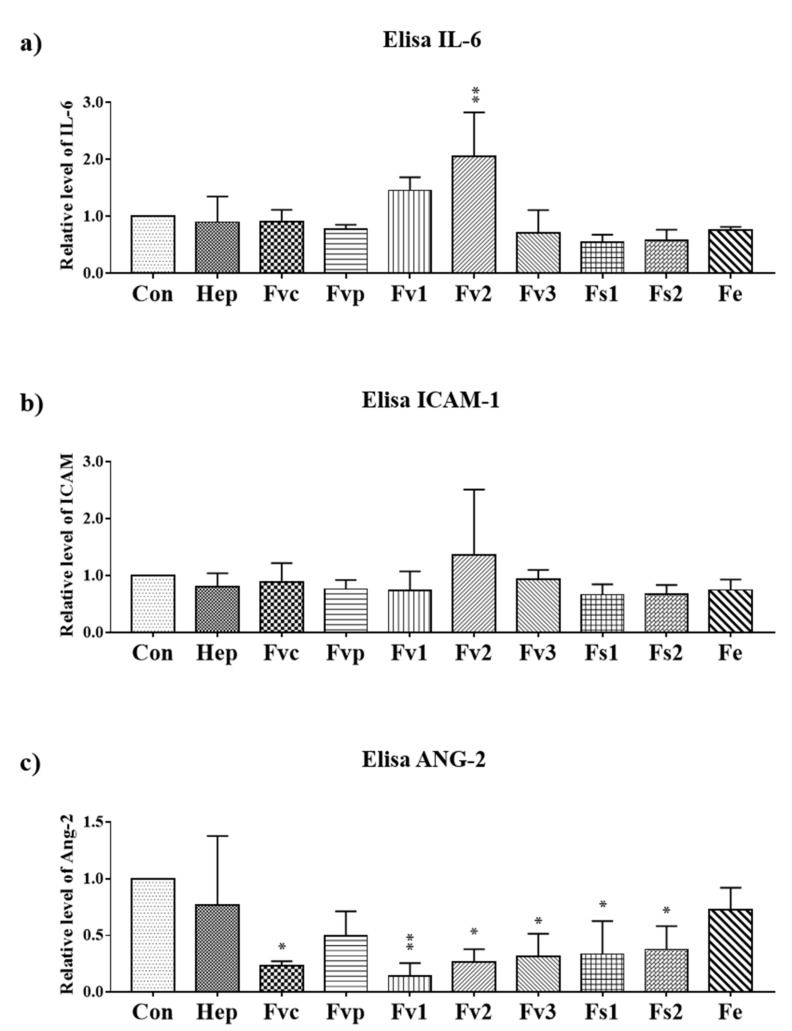
The protein level of inflammatory molecules (**a**) IL-6, (**b**) soluble ICAM-1 and (**c**) angiopoietin 2 (ANG-2) in the supernatant determined by ELISA for OEC treated with 100 µg/mL fucoidan. One-way ANOVA, * *p* < 0.05, ** *p* < 0.01, *n* = 3.

To study the impact of fucoidan extracts (100 µg/mL) on inflammatory and angiogenic endothelial cell activation [19,20], the secretion of associated inflammatory mediators (interleukin-6 IL-6 and intracellular adhesion molecule-1(ICAM-1)) and the proangiogenic molecule ANG-2 produced by endothelial cells [21] was determined by ELISA (Figure 4a–c). In addition, we performed real-time PCR for gene expression of IL-6, ICAM-1, and vascular cells adhesion molecule-1 (VCAM-1) (Figure S3) but we focus here on the protein level due to the higher physiological relevance. For protein assessment, all samples were harvested after seven days. Only Fv2 treatment resulted in a significant increase of IL-6 compared with the control, indicating an inflammatory reaction of the endothelial cells by Fv2, which was further underlined by a tentative increase in soluble ICAM-1. A similar but not significant increase of IL-6 was observed for Fv1. Further, the results indicate a significant reduction of angiopoietin 2 (ANG-2) for most extracts except for Fvp and Fe, indicating a reduction of this proangiogenic molecule produced by endothelial cells.

### 2.6. Effect of Fucus Extracts on Metabolic Activity and Cytotoxicity in Human Mesenchymal Stem Cells

**Figure 5:**
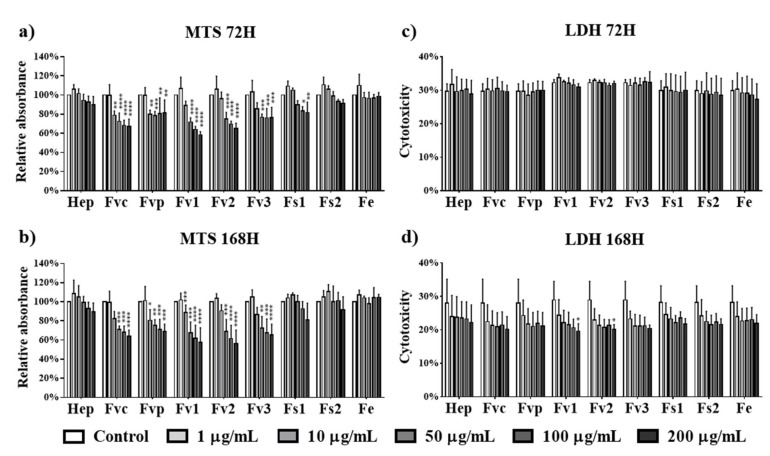
Cell metabolic activity of mesenchymal stem cells (MSCs) determined by MTS assay at (**a**) 72 h and (**b**) 168 h and cytotoxicity for MSCs determined by LDH at (**c**) 72 h and (**d**) 168 h after treated with fucoidan extracts and heparin at different concentrations of 1, 10, 50, 100, and 200 µg/mL. Two-way ANOVA, * *p* < 0.05, ** *p* < 0.01, *** *p* < 0.001, **** *p* < 0.0001, *n* = 3.

The effects of *Fucus* extracts on the metabolic activity and cytotoxicity for MSCs were tested using concentrations from 1 up to 200 µg/mL. In contrast to the results for endothelial cells, treatment with Fvc, Fvp, Fv1, Fv2, and Fv3 at concentrations higher than 10 µg/mL reduced the metabolic activity of MSC at both investigated time points (Figure 5a,b). For treatment with Fs1, only high concentrations of 100 and 200 µg/mL indicated a reduced metabolic activity after three days of incubation, while Fs2 and Fe extracts had no negative influence on the metabolic activity of MSC. Because LDH values as an indicator of membrane integrity (Figure 5c,d) were not significantly decreased, we assume that the decreased values in the MTS assay refer to a decrease in metabolic activity or proliferation rather than a cytotoxic effect.

**Figure 6:**
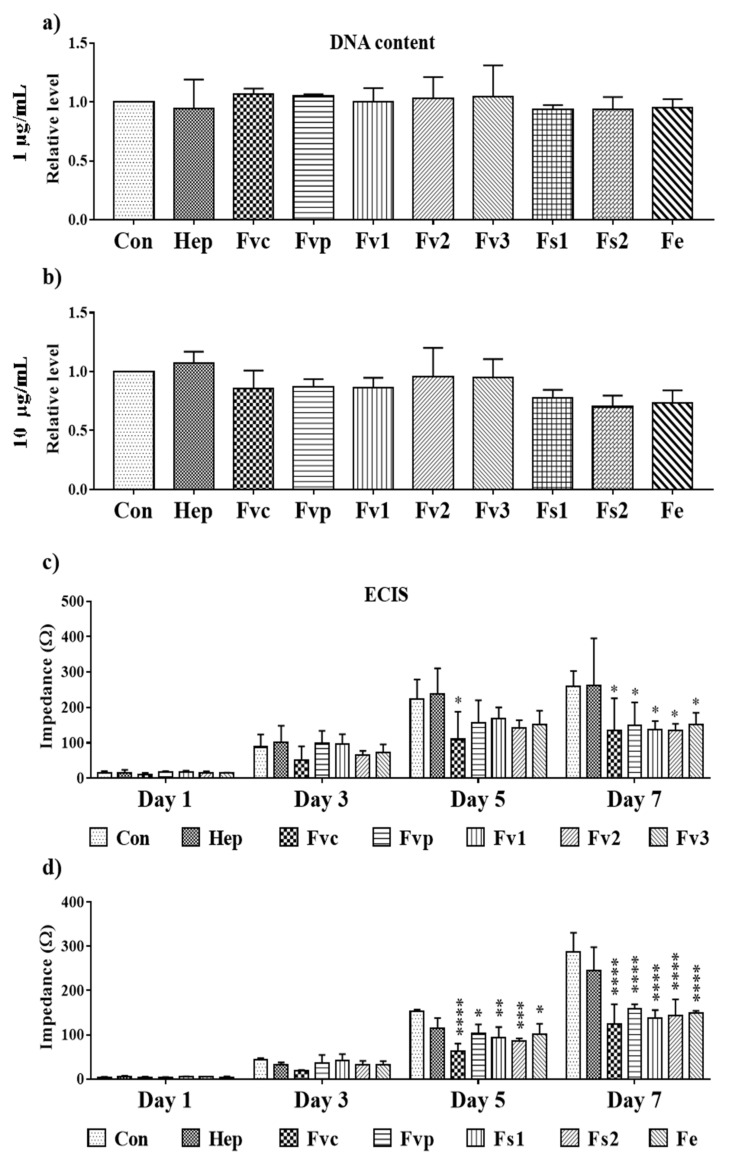
Effect of fucoidan on cell proliferation depicting the DNA content of MSC mono-cultures at (**a**) 1 and (**b**) 10 µg/mL fucoidan/heparin treatment and (**c**,**d**) impedance measurement for MSC (seeded at a density of 5000 cells/cm^2^) detected by ECIS at 100 µg/mL treatment. One-way ANOVA, * *p* < 0.05, ** *p* < 0.01, *** *p* < 0.001, **** *p* < 0.0001, *n* = 3.

To clarify, whether the MTS assay results reflect only a reduction of the metabolic activity or indicated a reduced proliferation, we investigated the effects using DNA quantification as a direct indicator of cellular proliferation at a concentration of 1 and 10 µg/mL. Results for DNA quantification at day 7 as depicted in Figure 6a,b did not show significant effects on MSC proliferation for the lower concentrations 1 and 10 µg/mL, although these concentrations reduced partly the metabolic activity. Nevertheless, the MSC seeding density in these experiments was relatively high, potentially interfering with the proliferation capacity by cell contact-mediated inhibition. Accordingly, we performed additional experiments using a lower cell seeding density and continuous ECIS impedance measurement over seven days, focusing on a concentration of 100 µg/mL. As depicted in Figure 6c,d, the impedance as an indicator of cell layer confluency showed a significant reduction for all tested Fucus extracts after seven days. Thus, tested *Fucus* extracts impaired the MSC proliferation using a higher concentration of 100 µg/mL and a lower cell density.

### 2.7. Impact of Fucus Extracts on the Osteogenic Activity in Human Mesenchymal Stem Cells

**Figure 7:**
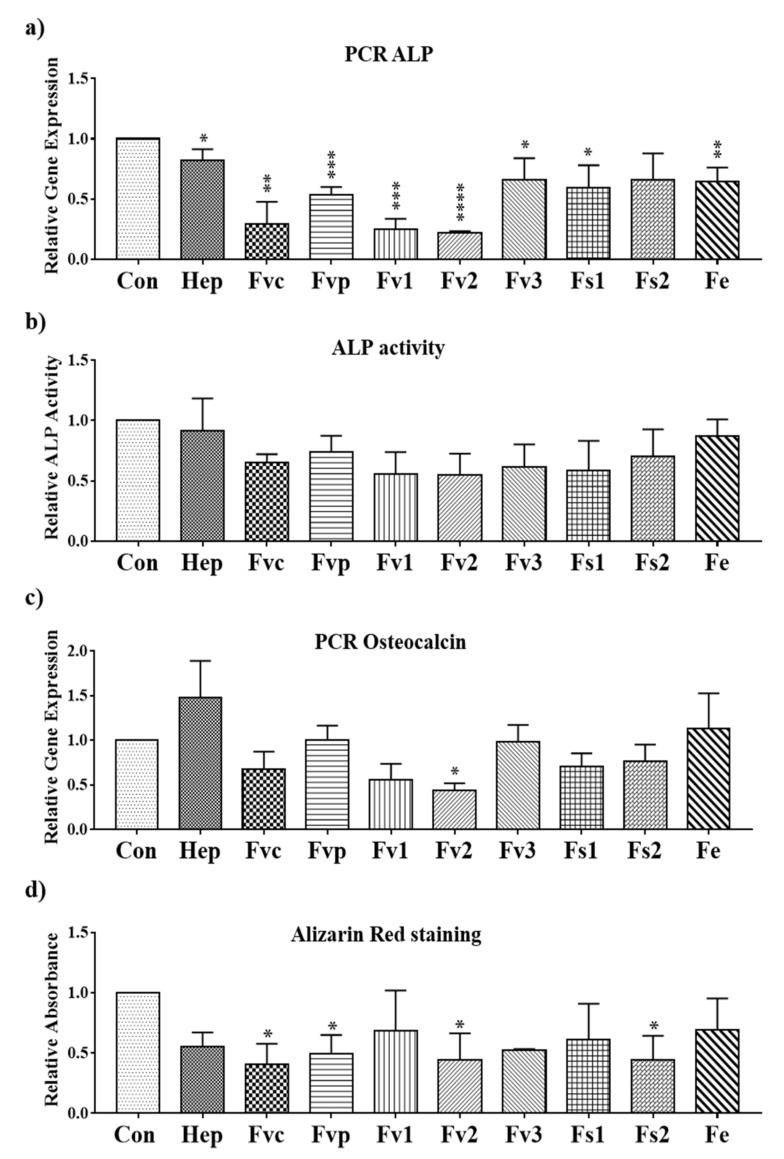
Relative gene expression for the osteogenic markers (**a**) ALP and (**c**) osteocalcin evaluated by semi-quantitative RT-PCR for MSC mono-cultures treated with fucoidan extracts and heparin at a concentration of 100 µg/mL on day 7; (**b**) ALP activity for the MSC mono-cultures with 100 µg/mL extracts treatment on day 14, and (**d**) the osteogenic activity determined by Alizarin Red staining for MSCs treated with 100 µg/mL extracts on day 14. One-way ANOVA, * *p* < 0.05, ** *p* < 0.01, *** *p* < 0.001, **** *p* < 0.0001, *n* = 3.

To evaluate if *Fucus* extracts from different species modulate the osteogenic differentiation of MSC, we determined the expression and activity of the early osteogenic differentiation marker alkaline phosphatase and the calcification process indicating the later stages of osteogenic differentiation. The gene expression of the early osteogenic marker alkaline phosphatase (ALP) was found to be significantly downregulated on day 7 for all the fucoidan treatments except for Fs2 (Figure 7a). On day 14, the ALP activity was lower in fucoidan-treated cells than in the untreated group, similar to PCR data, but no significant effect was observed (Figure 7b).

As shown in Figure 7d, the calcification level determined by Alizarin Red staining showed a significant reduction when MSCs were treated with Fvc, Fvp, Fv2, and Fs2. Other extracts had a similar but not significant effect on the calcification level, and results reflected widely data by PCR for osteocalcin (Figure 7c). 

### 2.8. Effects of Fucus Extracts on Regulatory Molecules for Angiogenesis in MSC 

As bioactive compounds with the potential to modulate angiogenesis in the bone, we investigated the effect of different fucoidan doses on key molecules such as vascular endothelial growth factor (VEGF) and angiopoietin 1 (ANG-1) expressed by MSC. We also studied the effect of fucoidan on stromal derived factor-1 (SDF-1), a chemokine that regulates the recruitment of various cell types including stem cells, osteoclast precursor cells, and immune-cells [22,23]. In previous studies, we found MSC to be the main producer of VEGF, SDF-1, and ANG-1 [24,25]. After fucoidan treatment, all tested extracts reduced the VEGF amount found in the supernatant of MSC (day 7). While Fvc, Fvp, Fv1, Fv2, and Fv3 caused a significant VEGF reduction at 10 µg/mL, Fs1, Fs2, and Fe treatment already lowered VEGF levels at the lowest tested concentration of 1 µg/mL. To achieve a significant reduction of SDF-1 levels in MSC, a concentration of 10 µg/mL was necessary for all tested extracts. Further, treatment with 10 µg/mL was necessary to reduce ANG-1 levels in MSC, with exception of Fe and Fs1, which significantly lowered ANG-1 already at the smallest tested concentration of 1 µg/mL. Additional data regarding the gene expression for angiogenic regulator molecules in OEC and MSC are depicted in Figure S4 (supplements). Overall, the protein data suggest a clear dose-dependent effect for all tested extracts and tested target molecules for MSC, although the concentrations required to lower the levels of these growth factors slightly varied depending on the extracts or the target molecule.

### 2.9. Influence of Selected Fucus Extracts on the Formation of Vascular Structures in MSC–OEC Co-Culture Models

To analyze the effect of fucoidan treatment on the formation of vascular structures on the functional level, we performed co-culture experiments that allow a direct interaction of endothelial cells and bone forming cells, resulting in the formation of microvessel-like structures. For this, OEC were co-cultivated with MSC as well as with the osteosarcoma cell line MG63 to model vascularization processes in both bone formation and bone tumors. 

**Figure 8:**
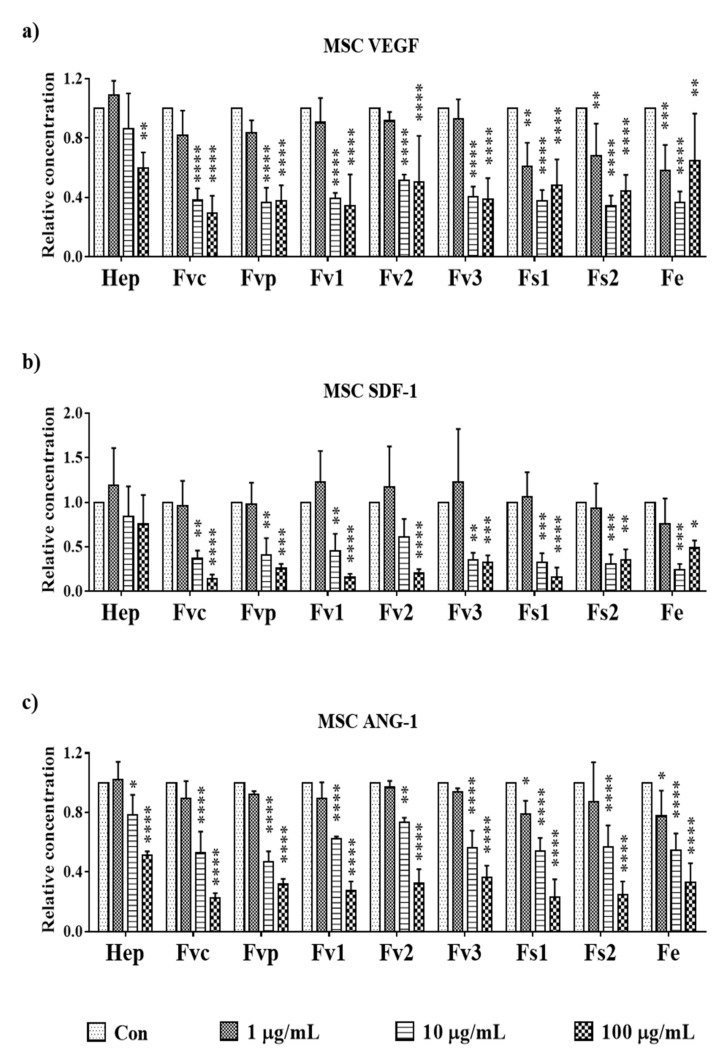
The relative amount of angiogenic growth factors: (**a**) vascular endothelial growth factor (VEGF), (**b**) stromal derived factor-1 (SDF-1), and (**c**) angiopoietin 1 (ANG-1) in supernatant of MSC mono-cultures treated with 100 µg/mL fucoidan/heparin on day 7, measured with enzyme-linked immunosorbent assay. One-way ANOVA, * *p* < 0.05, ** *p* < 0.01, *** *p* < 0.001, **** *p* < 0.0001, *n* = 3.

**Figure 9:**
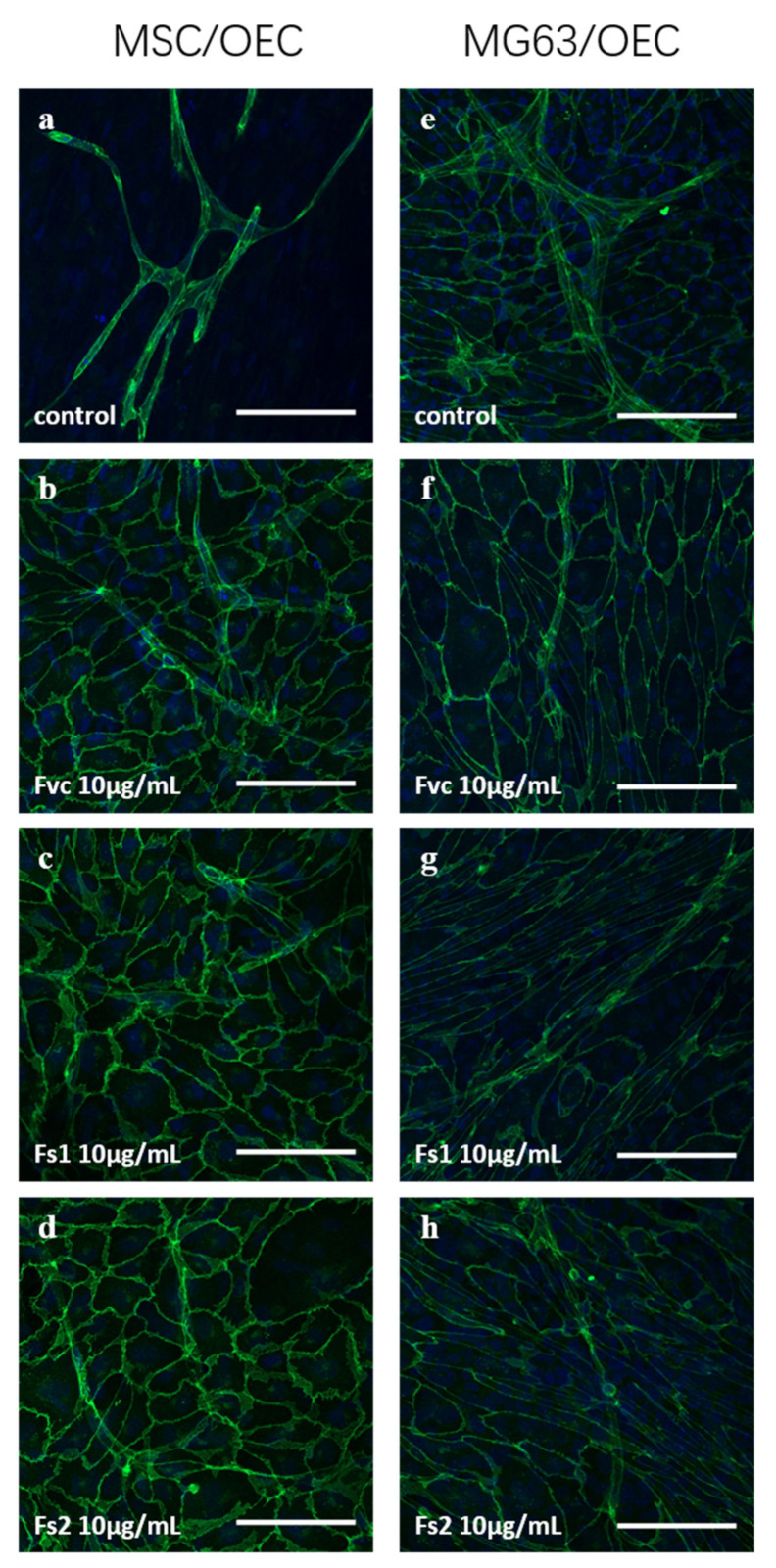
Confocal laser scanning microscopy for: MSC/OEC co-cultures on day 7 for control (**a**) and 10 µg/mL treatment of Fvc (**b**), Fs1 (**c**), and Fs2 (**d**); MG63/OEC co-cultures on day 7 for control (**e**) and 10 µg/mL treatment of Fvc (**f**), Fs1 (**g**), and Fs2 (**h**). Endothelial marker VE-cadherin is depicted in green, and nuclei are depicted in blue. The scale bar represents 100 μm.

Co-cultures were treated with selected *Fucus* extracts Fs1 and Fs2. In addition, Fvc was used as reference as it displayed antiangiogenic properties in our previous study [4]. Fs1 and Fs2 were selected due to a high tolerability for both cell types (Figure 1 and Figure 5), combined with a low inflammatory potential in endothelial cells but a high effectivity to modulate angiogenic molecules in OEC and MSC (Figure 4 and Figure 8). Both concentrations (10 and 100 µg/mL) of Fs1 and Fs2 lowered the number of vascular structures in both types of co-cultures, but results are depicted only for the lower concentration (Figure 9). These findings indicate an antiangiogenic effect of the extracts in accordance with their effects on the growth factors VEGF, ANG-1 and ANG-2, guiding the formation of vascular structures.

## 3. Discussion

Biomedical applications of compounds require solid data on biological activity and safety, including clear structure–function relationships. Crude fucoidan extracts are multicomponent products and their complex composition impede the structure–function correlations. As numerous studies have shown, the biological properties of fucoidans are highly dependent on their chemical properties, which in turn are tightly related to the species as well as the way they are harvested and extracted [11,26,27,28]. In this study, we evaluated six crude fucoidan extracts from three *Fucus* species obtained by different extraction methods. We compared them in terms of several aspects, starting with their chemical properties depending on the extraction procedure and species but mainly focusing on their impact on different cell types involved in bone formation and vascularization or the associated mediator molecules. The study provides a comprehensive data set by combining chemical characterization and biological assessment down to the cellular and molecular level using primary human cells.

Although this study does not specifically focus on a comparison of extraction methods, we noticed that extraction with 100 mM hydrochloric acid (HCl) led to pronounced loss of fucose and sulfate groups (Table 1). These data are in accordance with the literature, indicating that HCL-based extraction and higher acid concentrations interfere with the fucose or sulfate content or provoke degradation of polysaccharides in the extracts [29,30].

In contrast, the three extracts obtained by short-term heating with demineralized water and diluted (10 mM) sulfuric acid had higher fucose contents and DS values. The association between fucose content and DS was shown to be due primarily to the fact that the fucose units in fucoidans are sulfated [31]. In line with previous results [27,28], the treatment at 120 and 100 °C, respectively, was associated with degradation of the fucoidans, but not desulfation.

The tested fucoidan extracts largely varied in their mean MW as well as MW distributions (Table 1 andTable S1). With mean MW ranging between 84 kDa and 730 kDa and their wide MW ranges, they represent high molecular weight fucoidans in accordance with the common consent from literature. Although MW represents an important parameter for the biological activities of fucoidans [27,28], any conclusions regarding the impact of MW are limited by the very high polydisperity of the tested fucoidan extracts, which was even higher when HCl and sulfuric acid were used for extraction.

Products of natural origin may be contaminated with endotoxins. Therefore, we further analyzed the endotoxin content of the tested fucoidan extracts. Since endotoxins may cause inflammatory and immunological reactions [32,33], parentally applied medical products or materials getting in contact with blood must be endotoxin-free. Especially endothelial cells react highly sensitively toward endotoxins, with release of inflammatory molecules, changes in barrier, and increased angiogenic activity [34,35]. Thus, the evaluation of endotoxins was essential to avoid any artificial results and to exclude potential influences by endotoxins on the investigated parameters described in the following sections. 

Fucoidan extracts have differential effects on individual cell types, and their influences further vary in tumor-derived cell lines compared with primary cells [4]. In agreement with previous data from our group [4], endothelial cells compared with MSC tolerated higher doses of the tested fucoidans up to 200 µg/mL. Nevertheless, some of the tested extracts influenced the barrier and caused an increased secretion of IL-6, one of the main mediator molecules in inflammatory processes in endothelial cells [20]. If these observations correlate with the *fucus* species, the individual composition of the extracts or their extraction method cannot be finally clarified in this study. Nevertheless, such influences might correlate with the higher molecular weight, as indicated by the data for Fv1 and Fv2 in this study. 

Further, inflammation and changes in the barrier may also influence the formation of vascular structures [36]. Thus, these extracts were excluded in the functional assessment of angiogenesis in the co-cultures. 

In comparison with endothelial cells, MSC were more sensitive in response to fucoidan treatment. MTS values as indicators of metabolic activity were reduced at concentrations of 10 µg/mL or higher for the *Fucus vesiculosus* extracts. Similar results were reported before by our group for commercially available crude *Fucus vesciculosus* extracts. Although the extracts Fs1, Fs2, and Fe did not reduce the metabolic activity in MSC, all tested extracts impaired the proliferation of MSC at 100 µg/mL, indicated by ECIS measurement. We presently speculate that the reduced proliferation observed at higher doses might be associated with the auto-regulatory function of the growth factor VEGF. Previous publications have demonstrated an autocrine influence of VEGF on MSC proliferation and differentiation [37,38,39]. Thus, reduced VEGF levels, discussed in more detail in the next sections, may be responsible for impaired proliferation and metabolic activity of MSC.

Fucoidans have been reported to influence vascularization processes [40], although the detailed mechanisms are still not elucidated in detail [41] and even contradictory effects were described in the literature [42]. In our study, the influence of fucoidans on growth factors in the cell culture supernatants was determined using ELISA. All fucoidan extracts significantly lowered the concentrations of VEGF in the supernatants of MSC monocultures in a dose-dependent manner. These data indicate a reliable biological effect even in the context of a primary cell system for the bone, including donor-related variations in growth factor expression or non-homogeneous cell populations in MSC. For the extracts Fs1, Fs2, and Fe, a significant impact was already observed at 1 µg/mL, whereas for other extracts concentrations of 10 µg/mL were needed to achieve a significant reduction. For ANG-1, comparable significant effects were observed at the lowest tested dose, confirming the higher effectivity of Fs1 and Fe.

Interestingly, Fs1 and Fe were the extracts with the highest DS, followed by Fv3 and Fs2, potentially responsible for the impact on the VEGF observed at the lowest tested dose. The DS was shown to be an important factor for the pharmacological activities of fucoidans. In our previous study fucose and DS enriched fractions gained from *Fucus distichus* subsp. *evanescence* resulted in lower VEGF levels and increased the antiangiogenic properties of these fractions in comparison with their crude counterparts[15]. Thus, high fucose and DS seem to be key features for lowering VEGF levels, resulting in antiangiogenic effects. 

Beyond VEGF, which is mainly produced by MSC, ANG-2 produced by endothelial cells [24] is a critical co-factor guiding angiogenesis. The combination of VEGF and ANG-2 is needed to induce new vessel formation and angiogenic activation of endothelial cells [43]. In this study, all tested fucoidans besides Fe reduced ANG-2 produced by endothelial cells. Furthermore, ANG-2 is a factor in inflammation and tumor metastasis [44]. In addition, the chemokine SDF-1, involved in stem- and inflammatory cell recruitment [45], vascularization [46], and osteosarcoma metastasis [47,48], was consistently reduced by all tested extracts. 

Although effects on the angiogenic mediator are important parameters to indicate a therapeutical potential of fucoidans to control angiogenesis, vascularization processes are highly complex and dynamic and underlie a spatial and temporal control of multiple factors. Accordingly, evaluation of pro- or antiangiogenic properties of fucoidans should be supported by functional evaluation in a defined physiological context. For functional evaluation, we have chosen two co-culture models, allowing direct interactions between endothelial cells and bone cells [24,49]. In one, we used MSC to model bone formation; in the other one, we used the tumor cell line MG63 to model osteosarcoma. The selected extracts for these experiments, Fs1 and Fs2, showed antiangiogenic properties in both co-culture systems. The results were similar to previous reports using commercially available high molecular weight fucoidan [4]. The antiangiogenic effect in this present and in our previous study correlated with reduced concentrations of the growth factors VEGF, ANG-2, and ANG-1 in a very consistent and dose-dependent manner. All tested extracts resembled mainly HMW fucoidan but with variations in the molecular weight. A recent study by Gupta et al. showed antiproliferative effects of fucoidans on the osteosarcoma cell line MG63 depending on the species and molecular weight, resulting in the G1 arrest for high and medium molecular weight fucoidan treated cells [50]. Although MSC used in this present study proliferate more slowly compared to MG63, we observed to some extent similar effects for MSC, indicating that high molecular weight fucoidan may impair osteogenic cells’ metabolic activity and proliferation.

## 4. Materials and Methods

### 4.1. Fucoidan Extraction

*Fucus vesiculosus* (Fv), *Fucus* serratus (Fs), and *Fucus* subs. *evanescens* (Fe) were harvested in Kiel, Germany, and used for the extraction of crude fucoidan as described in detail by Ptak et al. [51]. All seaweeds were initially soaked in 85% ethanol solution overnight, followed by a wash with acetone. After drying, the fucoidans were extracted. The extracts Fs1 and Fs2 were extracted from *Fucus serratus* (harvested in October) with 10 mM sulfuric acid at 100 °C for 30 min (Fs1) and 100 mM hydrochloric acid at 80 °C (Fs2) for 30 min, respectively, using microwave-assisted extraction. The extracts were subsequently neutralized with 1 M sodium hydroxide and transferred to a conical tube. A strong solution (35%) of calcium chloride was added to the extracts to reach a final concentration of 1% in each tube. The extracts were then centrifuged (30 min at 4 °C), and the supernatant was transferred to a new conical tube. Ethanol (96%) was added to each tube for a final concentration of 40%. After the addition of ethanol, the extracts were centrifuged, and the supernatant was recovered. A final addition of ethanol was applied for a concentration of 70%, and the extracts were centrifuged a final time. The pellet was recovered and washed with ethanol and acetone and left to dry. After drying, the pellet was solubilized in demineralized water and dialyzed (MWCO = 12–14 kDa) until the conductivity of the surrounding water no longer increased. After dialysis, the extracts were freeze-dried and analyzed for chemical characterizations. For the following extracts, purification was performed as previously described unless stated otherwise. The crude fucoidan extract Fv1 was prepared by soaking *F. vesiculosus* (harvested in July) in 100 mM hydrochloric acid for 24 h, followed by neutralization with 1 M sodium hydroxide. Fv2 was similarly prepared as Fv1 from *Fucus vesiculosus* (harvested in October); however, the soaking and decanting step was repeated twice for three soaking steps in total. Fv3 was extracted from *Fucus vesiculosus* (harvested in October) with demineralized water at 120 °C for 30 min using microwave-assisted extraction. Fe was prepared from *Fucus distichus* subsp*. evanescens* (in July) and extracted similarly to Fv3. Specification of extracts and isolation are summarized in Table 3.

### 4.2. Chemical Characterization

#### 4.2.1. Elemental Analysis

The content of hydrogen, carbon, nitrogen, and sulfur was determined by elemental analysis as described previously [16]. The degree of sulfation (DS, number of sulfate group per monosaccharide) was calculated using the determined sulfur content (%) [16]. The total protein content was estimated by multiplying the nitrogen content (%) by 6.25.

#### 4.2.2. Monosaccharide Composition by GLC

To determine the neutral monosaccharide composition, acetylation analysis of the fucoidans was performed as previously reported [16]. Briefly, the fucoidans were hydrolyzed using 2M TFA [52], converted into alditol acetate derivatives (AA) by reduction and acetylation [18]. The AA derivatives were separated by gas-liquid chromatography (GLC), and the percentage of the respective AA was calculated.

#### 4.2.3. SEC-MALS-VIS Analysis

The molecular mass of the fucoidans was measured by size-exclusion chromatography (SEC) using an HPLC system equipped with multi-angle light scattering (MALS), viscometry (VIS) and refractive index (RI) detectors. The separation of the samples was achieved using an Agilent 1260 series quaternary pump with an OHPak LB-806M (8.0 mmID X 300 mmL; ShodexTM, Agilent, Waldbronn, Germany). The column outlet was connected to HELEOS-II MALS photometer (Wyatt Technology, Dernbach, Germany), followed by a ViscoStar III differential viscometer and an Optilab T-rEX differential refractometer (Wyatt Technology, Dernbach, Germany). The eluent was PBS buffer solution (50 mM Na_2_HPO_4_-NaH_2_PO_4_ and 150 mM NaCl, pH 7.0), and the flow rate was 0.7 mL/min. The fucoidan samples (2 mg/mL) were filtered (0.45 µm) before injection. The injection volume was 100 µL. The used dn/dc was 0.150 as previously described for fucoidans [17]. The data were collected and processed using the Astra (v.7.3.2) software (Wyatt Technology, Dernbach, Germany).

### 4.3. EndoLISA^®^ Endotoxicity Assay

Freeze-dried fucoidan extracts were solubilized at a concentration of 5 mg/mL in water and sterilized by sterile filtration through 0.2 µm filter. Only fully soluble extracts were used for the biological assessment.

EndoLISA^®^ kit (Hyglos, Germany) was used to determine the endotoxin content of the fucoidan samples according to the manufacturer’s instruction. The fucoidan samples were diluted to 100 µg/mL. Both samples and standards were prepared in duplicates and applied to the pre-treated lipopolysaccharide (LPS)-specific phage-binding plate, followed by adding the binding buffer and incubation overnight (18 h mixing at 450 rpm and 37 °C). After 3 washing steps to remove the matrix substances, assay reagent was applied. The fluorescence after 90 min was read at 380/445 nm of excitation/emission wavelength, and the relative fluorescence unit was calculated. The endotoxin content of fucoidan samples was calculated from a standard curve.

### 4.4. Ethical Approval for the Use of Human Cells

The MSC and OEC were isolated from human tissues. The use of human tissue and cells was approved by the local ethical advisory boards, including the consent from the individual donors. 

### 4.5. Isolation and Culture of Human Mesenchymal Stem Cells (MSCs)

Human mesenchymal stem cells (MSCs) were isolated from bone tissue fragments from femoral head as described by our group before [24,25]. In brief, bone marrow cells from cancellous bone structures were collected by washing bone fragments in tissue buffer (Medium 199), GlutaMaxTM (Gibco, Darmstadt, Germany), 20% fetal bovine serum (FBS) (Sigma, Taufkirchen, Germany), 1% penicillin/streptomycin (Pen/Strep) (Biochrom, Berlin, Germany), 1% fungizone (Biozol, Eching, Germany), and 1% ciprobay (FRESENIUS KABI, Bad Homburg, Germany). After centrifugation, cells were plated at a density of 2 × 10^6^ cells/cm^2^ to collagen type I (Corning, Bedford, MA, USA) coated flasks in Dulbecco’s Medium Essential Medium (DMEM)/Ham F-12 (Biochrom, Berlin, Germany) supplemented with 20% FBS and 1% Pen/Strep. Cells from passage 2 on cells were cultivated in osteogenic differentiation medium (ODM). ODM consisted of DMEM/Ham F-12, 0.1 μM dexamethasone (Sigma-Aldrich, St. Louis, MO, USA), 10 mM β-glycerol phosphate (Sigma-Aldrich, St. Louis, MO, USA), 50 μM ascorbic acid-2-phosphate (Sigma-Aldrich, St. Louis, MO, USA), 10% FBS, and 1% Pen/Strep. Cells were maintained for at least 2 weeks in ODM to obtain MSC with an osteogenic phenotype. Cells used in the presented study were in passage from 2 to 4 and were gained from MSC donors at the age of 78 (m), 69 (f), and 48 (m).

### 4.6. Isolation and Culture of Human Outgrowth Endothelial Cells (OECs)

Human outgrowth endothelial cells (OECs) from peripheral blood were isolated and cultured by the methods described previously [53,54]. Briefly, human mononuclear cells were isolated from buffy coat by gradient centrifugation using Biocoll (Biochrom, Berlin, Germany). After being resuspended in Endothelial Cell Growth Medium 2 (ECGM-2) (PromoCell, Heidelberg, Germany) with supplements from the kit, 5% FBS, and 1% Pen/Strep, cells were seeded in collagen type I coated 24-well plates at a density of 2.6 × 10^6^ cells/cm^2^ and further sub-cultured to new collagen type I coated 24-well plates after 7 days. Within 2–3 weeks, cobblestone-like OECs appeared, and cells were expanded for the experiments. Cells (passage 6–7) in the presented study were from buffy coat donors at the age of 59 (f), 25 (f), 20 (f), and 66 (m).

### 4.7. Cell Seeding and Fucoidan Treatment of Individual Cell Types 

MSC or OEC were seeded at a density of 40.000 cells/cm^2^ to multi-well plates coated with fibronectin for OEC monocultures and with collagen I (Corning, Badford, MA, USA) for MSC mono-cultures (Millipore, Temecula, CA, USA).

MSCs were cultured in ODM; OECs were maintained in ECGM-2. One day after seeding, the cells were treated with the fucoidan extracts described in Table 1 and commercial fucoidan extracts (Sigma, F5631-1G and F8190, Sigma, Taufkirchen, Germany) as well as heparin (physico-chemical analysis, Y0001282) at different concentrations in cell medium. The treatment was exchanged every 3 days along with medium exchange. 

### 4.8. MTS Cell Metabolic Activity Assay

The metabolic activity of cells was determined with CellTiter 96^®^ AQueous One Solution Cell Proliferation Assay (Promega, Madison, WI, USA). MSC or OECs were seeded on 96-well plates at a density of 40.000 cells/cm^2^. Cells were treated with concentrations of 1, 10, 50, 100, and 200 µg/mL fucoidan extracts and heparin. To characterize cell metabolic activity, the cells were incubated with MTS solution (1:6 diluted in medium) at 37 °C for 2 h. The absorbance at 490 nm was measured using a microplate reader. The cellular metabolic activity was calculated relative to the control group (100%) after subtracting the background absorbance. 

### 4.9. LDH Cytotoxicity Assay

To determine the cytotoxicity of fucoidan extracts and heparin in MSC or OEC at different concentrations, we performed LDH (lactate dehydrogenase) assay (Thermo Scientific, Rockford, IL, USA) at 24, 72, and 168 h after treatment using the cell culture supernatants as described in the previous section and the protocol according to manufacturer. First, the maximum LDH control was prepared by adding lysis buffer to cells in technical triplicates. After 45 min incubation at 37 °C, the supernatants were transferred to a 96-well plate and reaction mixture was added. After 30 min incubation at room temperature, the stop solution was applied, and the OD was measured at 490 and 680 nm on a plate reader. The absorbance at 680 nm was subtracted from the absorbance at 490 nm, and the cytotoxicity was calculated as a percentage relative to the maximum LDH activity control.

### 4.10. Immunofluorescence Staining and Visualization of OECs

OECs were seeded on fibronectin coated Thermanox coverslips (Thermo scientific, Rochester, NY, USA) in 24-well plates at a density of 40.000 cells/cm^2^. OECs were treated with fucoidan extracts for 7 days and fixed with 4% paraformaldehyde in phosphate buffered saline (PBS) (Affymetrix, Cleveland, OH, USA), followed by 3 times of a 5 min wash with PBS and 10 min permeabilization with 0.5% Triton^®^ X-100. Cells were incubated with VE-cadherin (R&D, Minneapolis, MN, USA) antibody (1:50 diluted in PBS with 1% bovine serum albumin (BSA)) for 1.5 h and with the secondary antibody (1% BSA in PBS) for 30 min after being washed with PBS 3 times for 5 min. Cells were incubated for 10 min with Hoechst (2 µg/mL in PBS). The cells on Thermanox coverslips were mounted with Fluoromount Aqueous Mounting Medium (Sigma-Aldrich, St. Louis, MO, USA) for subsequent visualization with confocal laser scanning microscopy (CLSM, LSM 510 Meta, Zeiss, Oberkochen, Germany).

### 4.11. Electrical Cell-Substrate Impedance Sensing (ECIS) for MSC and OEC 

OECs were seeded to 8W10E slides coated with fibronectin (Applied BioPhysics, Troy, NY, USA) at a density of 40.000 cells/cm^2^ in ECGM-2 medium. Then, cells were cultured for 3 days in ECGM-2 to reach 100% confluence and to establish a tight cell barrier before the cells were treated for 7 days with fucoidan and heparin while monitoring the impedance. The impedance as a percentage in relation to the control was analyzed. 

For MSC, cells were seeded at a lower density of 5000 cells/cm^2^ on the collagen I coated 8W10E slides in order to monitor the impedance as an indicator of MSC proliferation. The cells were treated the second day after seeding. The impedance values were recorded for 7 days with ECIS (applied biophysics, Troy, NY, USA). The mean values were calculated after subtracting blank values for each condition.

### 4.12. Quantification of DNA Content 

The double-stranded DNA content of MSC mono-cultures in 24-well plates was determined to analyze the impact of fucoidans on the cellular proliferation. DNA content was determined with Quant-iT PicoGreen dsDNA assay kit (Molecular probes, Eugene, OR, USA). To prepare the DNA aqueous solution, cells were trypsinized in 24-well plates. After centrifuging the cell suspension for 5 min at 2000 g, cell pellets were collected and resuspended in 1 mL deionized water. Cell membranes were ruptured to release DNA by three freeze–thaw cycles and two 15 s sonications. Samples and standards were prepared in triplicates. DNA amount was determined by measuring the fluorescence using a microplate reader (TECAN, Maennedorf, Switzerland) at 485/535 nm of excitation/emission wavelength.

### 4.13. Gene Expression Analysis

Total RNA isolation from MSCs and OECs was performed according to the manufacturer’s protocol using Total RNA Kit (VWR peqlab, Erlangen, Germany). The RNA concentration was determined with a NanoDrop (Thermo Fisher, Erlangen, Germany). Then, 1 µg of total RNA per sample was transcribed to cDNA with high capacity RNA-to-cDNA Kit (Applied Biosystems, Carlsbad, CA, USA). PCR was performed using primers as indicated in Table 4 using RPL13a as an internal control gene. Quantitative real-time PCR was carried out using a total volume of 20 µL for each reaction. The mastermix contained SYBR^®^ Select Master Mix (Applied Biosystems, Austin, TX, USA), cDNA QuantiTect^®^ Primer Assay (Qiagen, Hilden, Germany), RNase free water (Qiagen), and 3.2 µL cDNA. The mixtures were preheated to 50 °C for 20 min and 95 °C for 20 min, followed by 40 cycles of 95 °C for 15 s (step 1) and 60 °C for 60 s (step 2). The relative gene expression was calculated with the ΔΔcT method. Fucoidan or heparin treated groups were normalized to untreated controls. 

### 4.14. Enzyme Linked Immunosorbent Assay (ELISA)

To examine the protein levels in supernatant, the sample supernatants were collected to perform IL-6, ICAM-1, and angiopoietin 2 ELISA for OEC mono-cultures and, respectively, VEGF, SDF-1, and angiopoietin 1 ELISA for MSC mono-cultures using the Duo-Set ELISA Development kits (R&D, Minneapolis, MN, USA) according to the manufacturer’s protocols. The optical absorbance was detected by a microplate reader at 450 nm with a reference wavelength of 560 nm. The protein levels were presented relative to the controls. 

### 4.15. Quantitative Analysis of Osteogenesis

To quantitatively determine the influence of 100 µg/mL fucoidan on the calcification level in MSC mono-cultures at day 14, 1 mL of 40 mM Alizarin Red S Stain Solution (Millipore, Billerica, MA, USA) was applied to 4% PFA (paraformaldehyde) fixed cell monolayers in 24-well plates and incubated for 30 min. Cells were washed with distilled water until the wash solution became colorless and were incubated with 10% (*w*/*v*) cetylpyridinium chloride (CPC) (Roth, Karlsruhe, Germany) overnight to extract the Alizarin Red. Extracts were added to a 96-well plate containing standards prepared as well in 10% CPC. Absorbance of the samples was measured at a wavelength of 560 nm in a microplate reader.

### 4.16. Alkaline Phosphatase (ALP) Activity Assay

To measure the impact of fucoidan in MSC mono-cultures on ALP activity, the supernatant was collected 7 days after treating with 1, 10, and 100 µg/mL fucoidan, and an alkaline phosphatase assessment was performed according to the manufacturer’s protocol from the kit (Abcam, Germany). In brief, samples and standards were prepared in triplicates in 96-well plates for ALP reaction with pNPP Solution. Stop solution was added to the control wells before starting the ALP reaction to determine the background values. After 60 min incubation, the reaction in samples was stopped by stop solution, and the OD value was measured at 405 nm with a microplate reader. The values were calculated after background subtraction and are presented relative to the control.

### 4.17. Co-Cultures

MSC or the osteosarcoma cell line MG63 (ATCC, Wesel, Germany) were seeded at a density of 40.000 cells/cm^2^ on collagen I (Corning, Badford, MA, USA) coated Thermanox coverslips in 24-well plates. Then, OECs were added to MSC or MG63 cultures the second day in the same seeding density. The co-cultures were maintained in ECGM-2 and treated with 10 or 100 µg/mL fucoidan extracts (Fvc, Fs1, and Fs2) one day after seeding OECs. Cells were fixed with 4% PFA and stained on day 7 as described in 2.10.

### 4.18. Statistical Analysis

All experiments mentioned above were carried out with cells from at least 3 different donors. The statistical significance of the results was assessed with unpaired one-way ANOVA or two-way ANOVA using Graphpad Prism 7. As indicated in the corresponding result sections, *p* < 0.05 (* *p* < 0.05, ** *p* < 0.01, *** *p* < 0.001, **** *p* < 0.0001) was considered as statistically significant.

## 5. Conclusions

In conclusion, crude fucoidan extracts isolated from different fucus species represented mixtures of HMW fucoidans. Tested extracts consistently lowered important molecular mediators involved in angiogenesis in endothelial cells and MSC in vitro, but the doses needed to achieve a significant reduction differed amongst the extracts. In this context, the degree of sulfation seems to be a critical factor. The antiangiogenic effect was confirmed at the functional level in in vitro models mimicking vascularization in bone repair or bone tumors. Thus, HMW might be more useful in osteosarcoma treatment and less suitable for bone regeneration—something to be tested in in vivo models in the future.

## Figures and Tables

**Table 1:** Composition of neutral monosaccharides (Fuc (fucose), Xyl (xylose) Gal, (galactose), Glc (glucose)), degree of sulfation (DS), protein content, molecular mass, and rms radius of the extracted fucoidans from *fucus vesiculosus* (Fv), *fucus distichus* subsp. *evanescens* (Fe)and *fucus serratus (*Fs)

Monosaccharide Composition (mol%) ^a^					Degree of Sulfation ^b^ DS	Protein Content ^c^ (%)	Mw (kDa)	Rms Radius (nm)
	Fuc	Xyl	Gal	Glc				
Fv1	50.2	9.5	7.9	27.7	0.28	1.88	449	35
Fv2	57.7	18.5	13.2	2.9	0.26	1.08	730	33
Fv3	81.4	8.5	6.3	0.7	0.35	0.33	173	24
Fe	76.7	9.8	5.7	0.0	0.41	2.18	84	21
Fs1	76.2	6.5	3.3	11.2	0.61	0.52	272	41
Fs2	56.1	4.1	5.0	32.6	0.32	0.51	172	36

^a^ Determined according to the method of Blakeney et al. [18]. ^b^ Averaged number of sulfate groups per monosaccharide, calculated as -SO_3_Na residues from sulfur content determined by elemental analysis. ^c^ The content of protein was calculated by elemental analysis (nitrogen (%)).

**Table 2:** Endotoxicity of fucoidan extracts and heparin determined by EndoLISA^®^ kit.

Fucoidan Extracts/Heparin	Shortage	Endotoxicity (EU/mL)
Heparin (for physico-chemical analysis, Y0001282)	Hep.	0.0778
Fucoidan from *Fucus vesiculosus* crude (Fvc, Sigma, F5631-1G)	Fvc	0.0746
Fucoidan from *Fucus vesiculosus* pure (Fvp,Sigma, F8190)	Fvp	0.0750
Fv_KF_7-7-2017_SDU_24H_M1-0.1-HCL-22C_frac1	Fv1	0.0059
Fv_KF_10-10-17_SDU_24H_M1-22C_frac3	Fv2	0.0206
Fv_KF_170707_SDU_180405_M313D0.2	Fv3	0.0743
Fs_KF_171010_SDU_180501_M342D0.2	Fs1	0.0743
Fs_KF_171010_SDU_180405_M331D0.2	Fs2	0.0743
Fe_KF_170707_SDU_180405_M313D0.2	Fe	0.0732

**Table 3:** Comparison in fucoidan extraction for the 6 samples.

Ex.	Species	Harvest	Extracted		
			in	at	for
Fv1	*F. vesiculosus*	July	100 mM hydrochloric acid	RT	24 h
Fv2	*F. vesiculosus*	October	100 mM hydrochloric acid × 3	RT	24 h
Fv3	*F. vesiculosus*	October	demineralized water *	120 °C	30 min
Fe	*F. evanescens*	July	demineralized water *	120 °C	30 min
Fs1	*F. serratus*	October	10 mM sulfuric acid *	100 °C	30 min
Fs2	*F. serratus*	October	100 mM hydrochloric acid *	80 °C	30 min

Ex.: extracts; * microwave assisted; RT: room temperature.

**Table 4:** Primer List for individual molecules and internal housekeeping gene (RPL13A).

Gene Name	Primer Assay	Catalogue No.
ALP	Hs_ALPL_1_SG QuantiTect Primer Assay	QT00012957
Angiopoietin 1	Hs_ANGPT1_1_SG QuantiTect Primer Assay	QT00046865
Angiopoietin 2	Hs_ANGPT2_1_SG QuantiTect Primer Assay	QT00100947
Osteocalcin	Hs_BGLAP_1_SG QuantiTect Primer Assay	QT00232771
ICAM	Hs_ICAM1_1_SG QuantiTect Primer Assay	QT00074900
IL-6	Hs_IL6_1_SG QuantiTect Primer Assay	QT00083720
SDF-1	Hs_CXCL12_1_SG QuantiTect Primer Assay	QT00087591
VCAM-1	Hs_VCAM1_1_SG QuantiTect Primer Assay	QT00018347
VEGF	Hs_VEGFA_2_SG QuantiTech Primer Assay	QT01036861
RPL13A	Hs_RPL13A_1_SG QuantiTect Primer Assay	QT00089915

## Data Availability

Data is contained within the article or supplementary material.

## Supplementary Materials

The following are available online at https://www.mdpi.com/article/10.3390/md19040194/s1. Figure S1: Molecular mass-versus-elution time plots for (A) three fucoidans from F. vesiculosus (Fv1, Fv2 and Fv3) and (B) two fucoidans from F. serratus (Fs1 and Fs2) as well as one from F. distichus subsp. evanescens (Fe). RI chromatogram is overlayed in all plots, Figure S2: (A) Differential molecular mass distribution and (B) cumulative molecularar mass distribution analyses of three fucoidans from F. vesiculosus (Fv1, Fv2 and Fv3), two fucoidans from F. serratus (Fs1 and Fs2), and one from F. distichus subsp. evanescens (Fe). Differential distribution plot is the visual representatio of multimodal (more than one peak) or monomodal composition of the sample, while the cumulative distribution plot shows the weight fraction of certain molecular mass range, Figure S3. Relative gene expression for the inflammatory response relevant molecules a) IL-6, b) ICAM-1 and c) VCAM evaluated by semi-quantitative RT-PCR for mono-cultures of OEC treated with 100 µg/mL fucoidan on day 7. 1-way ANOVA. * *p* < 0.05, ***p* < 0.01. n = 3, Figure S4. Relative gene expression for the angiogenic molecules a) VEGF, b) SDF-1, c) ANGPT-1 for MSCs and d) ANGPT-2 for OECs with 100 µg/mL fucoidan and Heparin treatment evaluated by semi-quantitative RT-PCR on day 7. 1-way ANOVA. **p* < 0.05, ** *p* < 0.01. n = 3.
