# Supplementary material for: Influence of Fucoidan Extracts from Different Fucus Species on Adult Stem Cells and Molecular Mediators in In Vitro Models for Bone Formation and Vascularization
Source: Mar Drugs. 2021 Mar 29;19(4):194. doi: 10.3390/md19040194 (PMC8066524; doi:10.3390/md19040194)
Supplement: Supplementary file 1 [file marinedrugs-19-00194-s001.pdf]

Influence of Fucoidan Extracts from Different Fucus Species on Adult Stem Cells and Molecular Mediators in In Vitro Models for Bone Formation and Vascularization

Fanlu Wang [1], Yuejun Xiao [1], Sandesh Neupane [2,†], Signe Helle Ptak [3,†], Ramona Römer [1], Junyu Xiong [1], Julia Ohmes [1], Andreas Seekamp [1], Xavier Fretté [3], Susanne Alban [2] and Sabine Fuchs [1,*]

[1] Experimental Trauma Surgery, Department of Orthopedics and Trauma Surgery, University Medical Center Schleswig-Holstein, 24105 Kiel, Germany; fanluwang@gmail.com (F.W.); xiao_yuejun@163.com (Y.X.); ramona.roemer@googlemail.com (R.R.); serene.xj@gmail.com (J.X.); Julia.Ohmes@uksh.de (J.O.); Andreas.Seekamp@uksh.de (A.S.)

[2] Department of Pharmaceutical Biology, Pharmaceutical Institute, Kiel University, 24148 Kiel, Germany; sneupane@pharmazie.uni-kiel.de (S.N.); salban@pharmazie.uni-kiel.de (S.A.)

[3] SDU Chemical Engineering, University of Southern Denmark, 5230 Odense, Denmark; sihp@igt.sdu.dk (S.H.P.); xavier.frette@gmail.com (X.F.)

* Correspondence: Sabine.Fuchs@uksh.de; Tel.: +49-431-500-24561

† Equally contributed.

Table S1. Molecular mass ranges (≤ 20%, 20-80%, ≥ 80% of the weight fraction) of the six fucoidans. The data were calculated using the "distribution analysis" function of the Astra 7.3 software.

**Sample**	**MW range (kDa) of**		
	**≤ 20% (wt. fr.)**	**20-80% (wt. fr.)**	**≥ 80% (wt. fr.)**
Fv1	32-37	37-643	643-2812
Fv2	120-194	194-1344	1344-2812
Fv3	51-67	67-251	251-609
Fe	22-24	24-117	117-403
Fs1	9-118	118-477	477-1444
Fs2	6-10	10-258	258-1076

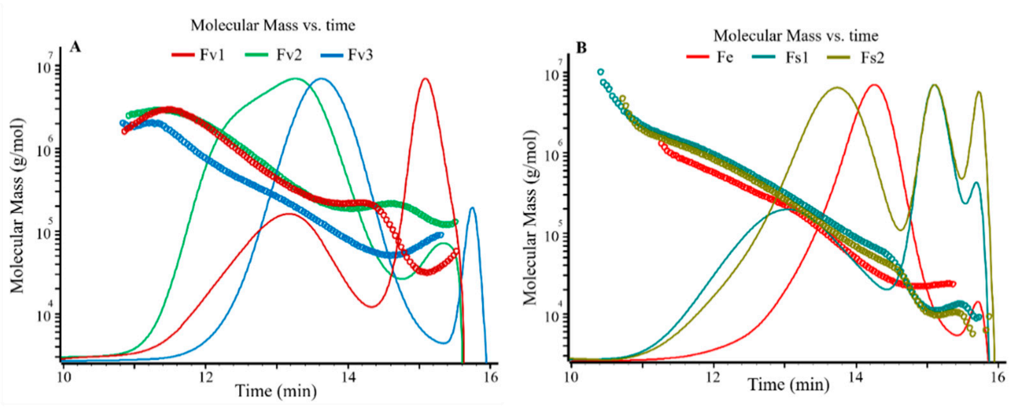

Figure S1: Molecular mass-versus-elution time plots for (**A**) three fucoidans from *F. vesiculosus* (Fv1, Fv2 and Fv3) and (**B**) two fucoidans from *F. serratus* (Fs1 and Fs2) as well as one from *F. distichus* subsp. *evanescens* (Fe). RI chromatogram is overlayed in all plots.

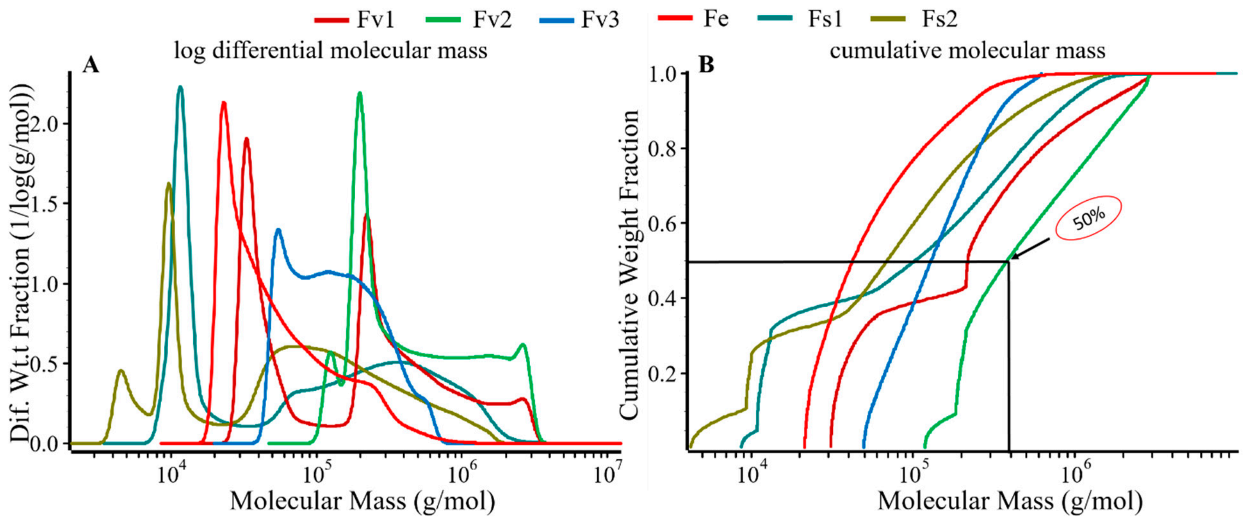

Figure S2: . (**A**) Differential molecular mass distribution and (**B**) cumulative moleculararar mass distribution analyses of three fucoidans from *F. vesiculosus* (Fv1, Fv2 and Fv3), two fucoidans from *F. serratus* (Fs1 and Fs2), and one from F. *distichus* subsp. *evanescens* (Fe). Differential distribution plot is the visual representatio of multimodal (more than one peak) or monomodal composition of the sample, while the cumulative distribution plot shows the weight fraction of certain molecular mass range.

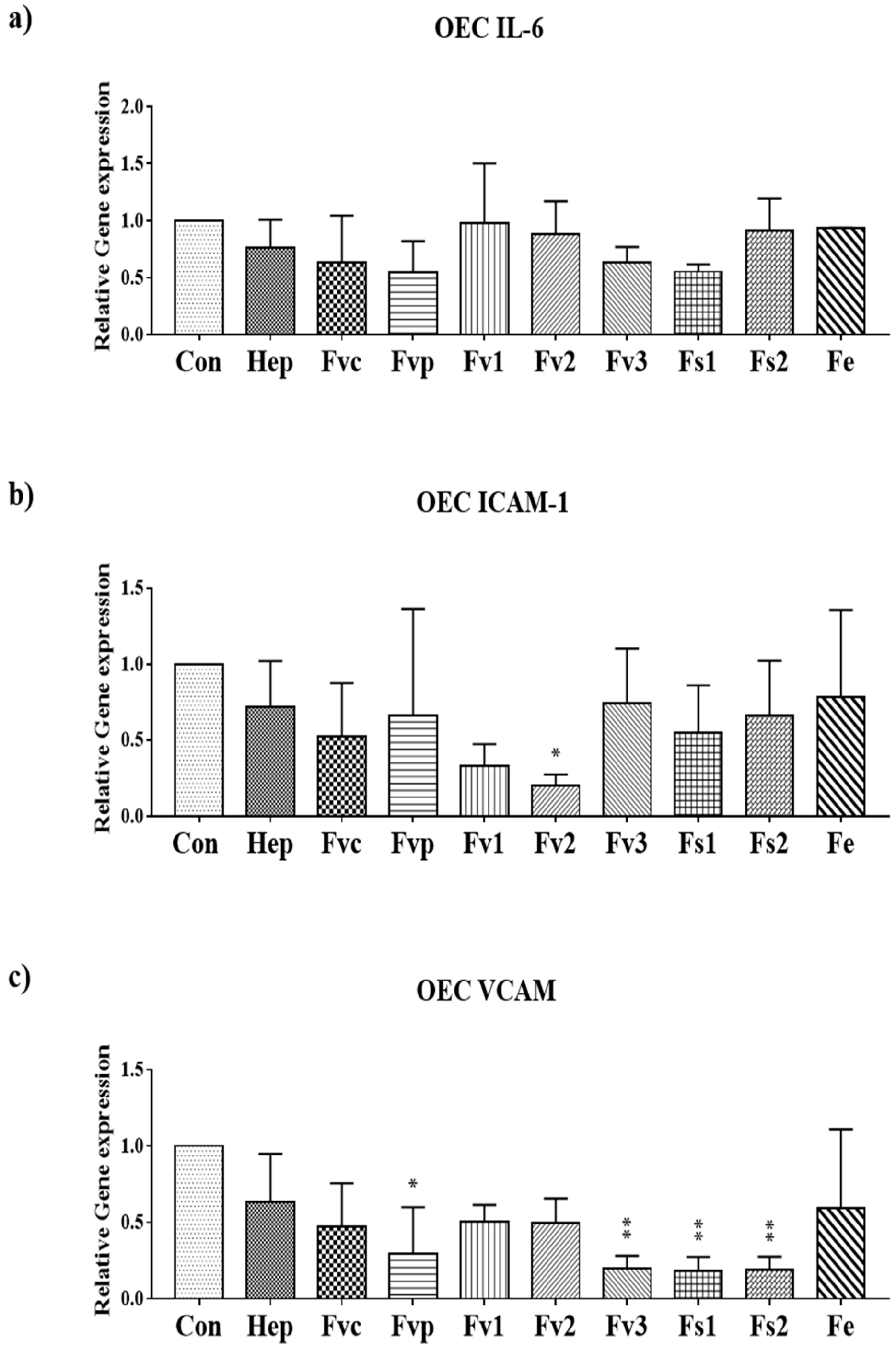

Figure S3. Relative gene expression for the inflammatory response relevant molecules **a**) IL-6, **b**) ICAM-1 and **c**) VCAM evaluated by semi-quantitative RT-PCR for mono-cultures of OEC treated with 100 μg/mL fucoidan on day 7. 1-way ANOVA. *p<0.05, **p<0.01. n=3.

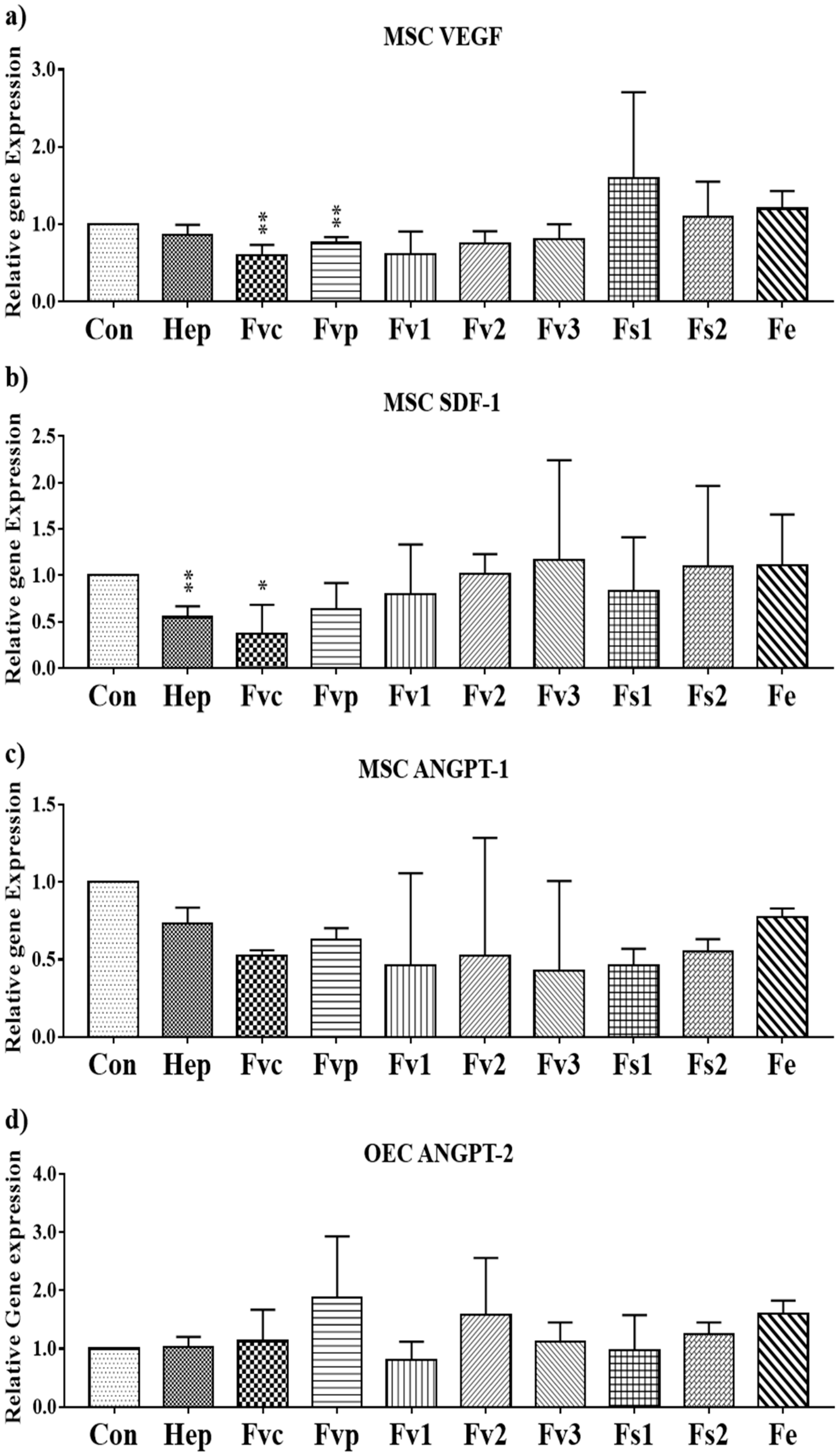

Figure S4. Relative gene expression for the angiogenic molecules **a**) VEGF, **b**) SDF-1, **c**) ANGPT-1 for MSCs and **d**) ANGPT-2 for OECs with 100 μg/mL fucoidan and Heparin treatment evaluated by semi-quantitative RT-PCR on day 7. 1-way ANOVA. *p<0.05, **p<0.01. n=3.
